# RETRACTED: Malenković et al. Resilience and Perceived Social Support in Cancer Survivors: Validity, Levels, and Sociodemographic Correlates of CD-RISC-25 and MSPSS Scales. *Healthcare* 2025, *13*, 1747

**DOI:** 10.3390/healthcare13182308

**Published:** 2025-09-16

**Authors:** Goran Malenković, Jelena Malenković, Sanja Tomić, Silvija Lučić, Armin Šljivo, Fatima Gavrankapetanović-Smailbegović, Slobodan Tomić

**Affiliations:** 1 Faculty of Medicine, University of Novi Sad, 21000 Novi Sad, Serbia; goran.malenkovic@mf.uns.ac.rs (G.M.); jelena.trivkovic@gmail.com (J.M.); sanja.tomic@mf.uns.ac.rs (S.T.); silvjia.lucic@mf.uns.ac.rs (S.L.); 2 Clinical Center of University of Sarajevo, 71000 Sarajevo, Bosnia and Herzegovina; sljivo95@windowslive.com (A.Š.); fatima.smailbegovic@gmail.com (F.G.-S.)

The journal retracts the article titled “Resilience and Perceived Social Support in Cancer Survivors: Validity, Levels, and Sociodemographic Correlates of CD-RISC-25 and MSPSS Scales” [1], cited above.

Following publication, the authors contacted the Editorial Office to raise concerns that due to an unintentional error, incorrect datasets were used in the publication [1].

Adhering to our standard procedure, the Editorial Office and Editorial Board conducted an investigation that confirmed that while applying the Bonferroni correction, the authors inadvertently combined statistical analyses from two different studies. As a result, the Editorial Office, Editorial Board, and authors have decided to retract this article as per MDPI’s retraction policy (https://www.mdpi.com/ethics#_bookmark30 (accessed on 3 September 2025)).

This retraction was approved by the Editor-in-Chief of the *Healthcare* journal.

The authors agreed to this retraction.

## References

[1] Malenković G., Malenković J., Tomić S., Lučić S., Šljivo A., Gavrankapetanović-Smailbegović F., Tomić S. (2025). RETRACTED: Resilience and Perceived Social Support in Cancer Survivors: Validity, Levels, and Sociodemographic Correlates of CD-RISC-25 and MSPSS Scales. Healthcare.

